# Clinical bioinformatics: a new emerging science

**DOI:** 10.1186/2043-9113-1-1

**Published:** 2011-01-20

**Authors:** Xiangdong Wang, Lance Liotta

**Affiliations:** 1 Department of Respiratory Medicine, Biomedical Research Center, Fudan University Zhongshan Hospital, Shanghai, PR China; 2 Center for Applied Proteomics and Molecular Medicine, George Mason University, Manassas, VA USA

## 

Welcome to the open-access journal titled *Journal of Clinical Bioinformatics (JCBi)*, a truly international journal devoted to clinical applications of bioinformatics, medical informatics and the development of bioinformatics tools, methodologies and approaches for clinical research. *JCBi *aims to discover how biological and medical informatics can be applied to the development of personalized healthcare, medication and therapies. The field of clinical bioinformatics includes the analysis of human microarray and other omics data, combination of bioinformatics and medical informatics, development of bioinformatics methodologies for clinical research, and human databases. *JCBi *also aims to establish a scientific channel to translate bioinformatics to clinical and medical application in order to better understand molecular and cellular mechanisms and therapies for human diseases.

Clinical bioinformatics is a new emerging science combining clinical informatics, bioinformatics, medical informatics, information technology, mathematics, and omics science together. At the beginning of the 20^th ^century, clinical physicians needed to be informed and open to advances in omics technology despite the barriers which existed for physicians applying genetic tests, for example the low tolerance for uncertainty, negative attitudes about their responsibility for genetic counseling and testing, and unfamiliarity with ethical issues raised by testing [1]. Since the middle of the 20^th ^century, bioinformatics was suggested to be applied for clinical toxicology [2] and cancer [3]. One of the early studies on expressed sequence tags in human stem cells by bioinformatics was performed in 1998 [4], where near 10000 sequences were analyzed. Of these, 48% showed the identity to known genes in the GenBank database, 26.4% matched to the previously deposited in a public domain database, 14% were previously undescribed sequences, and the remaining 12% were mitochondrial DNA, ribosomal RNA, or repetitive sequences. At the beginning of the 21^st ^century, gene expression profiles in 60 human cancer cell lines used in a drug discovery screen were evaluated by cDNA microarrays and corrected with drug activity patterns by combining bioinformatics and chemoinformatics [5]. Clinical bioinformatics was initially proposed to provide biological and medical information for individualized healthcare, enable researchers to search online biological databases and use bioinformatics in medical practice, select appropriate software to analyze the microarray data for medical decision-making, optimize the development of disease-specific biomarkers, and supervise drug target identification and clinical validation [6].

Clinical bioinformatics plays an important role in a number of clinical applications, including omics technology, metabolic and signaling pathways, biomarker discovery and development, computational biology, genomics, proteomics, metaboliomics, pharmacomics, transcriptomics, high-throughput image analysis, human molecular genetics, human tissue bank, mathematical medicine and biology, protein expression and profiling and systems biology. Understanding the interaction between clinical informatics and bioinformatics is the first and critical step to discover and develop the new diagnostics and therapies for diseases. Clinical bioinformatics was suggested to be associated with the analysis and visualization of complex medical datasets [7]. Different from other informatics, clinical bioinformatics should focus more on clinical informatics, including patient complaints, history, therapies, clinical symptoms and signs, physician's examinations, biochemical analyses, imaging profiles, pathologies and other measurements. It was emphasized that the simultaneous evaluation of clinical and basic research could improve medical care, care provision data, and data exploitation methods in disease therapy and algorithms for the analysis of such heterogeneous data sets [8]. This particular study tried to match disease complexity of patient information, clinical data, standard laboratory evaluations, brain imaging data and genetic data obtained from molecular profiling experiments. It is a huge difficulty and challenge to compel the clinical and biomedical data generated with bioinformatics from omics analyses. Clinical bioinformatics failed to show the importance, significance and clear relationships between clinical observations and the underlying molecular mechanisms due to the lack of integrated analysis and digitalized informatics of clinical descriptions and measurements. Thus, there is a great need for a scientific channel and platform like *Journal of Clinical Bioinformatics*, to exchange information on the development, standardization, application, and optimization of clinical bioinformatics for informaticists, bioinformaticsts, cellular and molecular biologists, pharmacologists, and clinicians.

Clinical bioinformatics is a new way to focus on the combination of clinical measurements and signs with human tissue-generated bioinformatics, understand clinical symptoms and signs, disease development and progress, and therapeutic strategy, and map relationships that integrate discrete elements that collectively direct global function within a particular -omic category, with clinical examinations, pathology, biochemical analysis, imaging and therapies. The *JCBi *perspective allows inspection and prediction of disease conditions, not limited to a monogenic challenge, but as a combination of individualized molecular permutations acting in concert to affect a phenotypic outcome. Bioinformatic integration of multidimensional data within and between molecular biology and medicine thus harbors the potential to identify unique biological signatures, providing an enabling platform for advances in clinical and translational science. There is a great need to have a special communication platform for both bioinformatics scientists and clinicians to exchange their knowledge and experience on the development of new biotechnologies, gene and protein functions, cell and organ dysfunction, and pathology, related to clinical signs, symptoms, findings, measures, prognosis and therapeutic effects.

The term "*Clinical bioinformatics*" is defined here as "clinical application of bioinformatics-associated sciences and technologies to understand molecular mechanisms and potential therapies for human diseases", a new and important concept for the development of disease-specific biomarkers, mechanism-oriented understanding and individualized medicine. There is solid evidence that the generation and expansion of genomic, transcriptomic, and proteomic data from human studies by high-throughput biotechnologies have increased enormously. In parallel, clinical measurements and examined information are elevated by the development of advanced clinical devices. Acquisition of high-dimensional datasets to combine both clinical and biomedical information and outcomes requires a communication platform as archival systems that permit efficiency of storage and retrieval. Multiple electronic repositories have been initiated and maintained. The number of published scientific papers related to "Clinical bioinformatics" significantly increases every year. *JCBi *provides a forum for exchange of ideas on potential molecular and cellular mechanisms, biomarker identification and validation, and drug discovery and development by the application of clinical bioinformatics. *JCBi *will also aim to play an important, critical, and recognized role in the improvement of understanding molecular mechanisms of diseases and development of new medicines. In addition, the journal is directed toward those specialists who work with disease-related bioinformatics, mathematics, biostatistics and molecular biology, those who explore drug discovery and development, pharmacology and toxicology, and pharmaceutical science, those who treat patients in the clinic and develop individualized medicine, and those who investigate molecular and cellular mechanisms involved in the development and reversibility of epithelium-involved diseases.

There is an urgent and immediate need to create a forum to stimulate discussion and exchange of scientific findings and understandings of clinical bioinformatics with a clear goal of treating diseases and improving the quality of patients. *JCBi *is the only journal focusing on the clinical application of bioinformatics and keeping track of the wealth of new information related to this topic. This is particularly the case when we observe the rapid development of new biotechnologies, e.g. genomics, proteomics, and celleomics, and the increasing capacities of clinical investigations. We believe that the *JCBi *will play an important, critical, and recognized role in understanding the molecular mechanisms of the diseases and developing the individual medicine and therapeutic strategy.

*JCBi *is also proud to be affiliated with the newly established International Society of Translational Medicine (ISTM) [9] and will be a prominent publication for its Omics Science section. As a non-profit organization, ISTM is a network of clinicians and researchers from all science fields with an interest in translational medicine. The partnership between *JCBi *and ISTM will assist with the interdisciplinary research across bioinformatics and translational medicine.

In conclusion, we as editors of *JCBi*, are delighted to welcome you to this new and novel journal and thank the scientists who have agreed to publish in the journal. In setting up the journal, we owe an enormous debt of gratitude to all professors and scientists for their encouragement, support, comments, suggestions, and contributions. With great support from our Associate Editors and Editorial Board Members [10], we deeply believe that *JCBi *will be well-received both by preclinical, clinical and pharmaceutical scientists interested in clinical bioinformatics and contribute to better outcome for understanding the diseases and developing new therapies. Involvement and contributions from a large group of scientists who work on clinical bioinformatics are crucial to the success of the journal.

Xiangdong Wang MD, PhD

Lance Liotta, PhD

Co-Editors-in-Chief

Journal of Clinical Bioinformatics
